# Fiber Post Removal Using a Conservative Fully Guided Approach: A Dental Technique

**DOI:** 10.1155/2022/3752466

**Published:** 2022-07-22

**Authors:** Abdullah Alfadda, Abdulmohsen Alfadley, Ahmed Jamleh

**Affiliations:** ^1^Endodontic Division, Dental Services, Central Region, King Abdulaziz Medical City, Ministry of National Guard Health Affairs, Riyadh, Saudi Arabia; ^2^Department of Restorative and Prosthetic Dental Sciences, College of Dentistry, King Saud Bin Abdulaziz University for Health Sciences, King Abdullah International Medical Research Center, Ministry of National Guard Health Affairs, Riyadh, Saudi Arabia

## Abstract

This report describes the usefulness of an endodontic template for the removal of a fiber post. A 40-year-old man presented with discomfort in the maxillary left canine. Clinical and radiographic examinations showed tooth #23 with a permanent core material retained with fiber post along with a persistent apical radiolucency. Among the various treatment modalities, nonsurgical root canal retreatment with fiber post removal using a conservative fully guided approach was proposed. After obtaining both the cone-beam computed tomographic images and the cast surface scan, their data were merged using implant planning software (ImplaStation for Windows x64 Bit Beta Version, ProDigiDent, Miami, Florida, USA) and superimposed. The drilling space was planned based on the location, diameter, and apical extent of the fiber post and was virtually overlapped and transferred clinically using a resin template to drill through the fiber post. With guides in position over the rubber dam, drilling was made with increments of 2 mm using a size 4 long-shank round bur (Thomas, Bourges, France) until it exposed the coronal gutta-percha. As soon as the canal was located, K3 rotary files (Sybron Endo, Orange, USA) were used along with chloroform to remove the old obturating materials. Then, additional shaping and cleaning were done with ProTaper Next rotary files (Dentsply Sirona, Ballaigues, Switzerland), sizes X2 and X3, and 5.25% NaOCl irrigation, respectively. The root canal was then dried with paper points and obturated with gutta-percha and AH Plus sealer (Dentsply Sirona, Ballaigues, Switzerland) using the continuous-wave compaction technique. Finally, the tooth was temporarily restored using the double seal technique with zinc oxide and zinc sulfate-based temporary material (Cavit W; 3M ESPE, St. Paul, MN, USA) and resin-modified glass ionomer material (Photac Fil; ESPE, Norristown, PA, USA) filling materials and referred for the final restoration.

## 1. Introduction

Recurring periapical pathology can develop after inadequate nonsurgical root canal treatment. A common procedure for clinicians to encounter is the retreatment of endodontically treated teeth with posts [1]. Metal posts retained with traditional types of cement such as zinc phosphate can usually be removed; however, in recent years, adhesively bonded glass [2], carbon [3], or quartz fiber [4] posts have become popular, replacing metal posts. The fiber posts are bonded into the root canal space with adhesive materials such as composite resins or glass ionomers, which are reported to be more difficult to remove [5, 6]. It is reported that the fiber posts can be fragmented and removed by using a microscope along with drilling with long-shank round burs, ultrasonic tips, and/or special removal kits [7, 8]. Nevertheless, currently used post removal techniques frequently result in procedural errors such as excessive removal of intraradicular dentin, deviation from the root axis, and perforation of the root structure [9]. Furthermore, these techniques are time-consuming and dependent on the clinician's experience [7, 9–12]. Post removal requires fragmentation within a limited anatomic area that is difficult to visualize, which may result in excessive substance loss leading to iatrogenic errors that compromise stability and hence compromise the tooth prognosis [13].

Cone-beam computed tomographic (CBCT) imaging has been recently recommended in endodontics as a diagnostic aid in root canal treatment planning [14, 15]. It lays the foundation for the 3D printing of endodontic templates [16]. In guided endodontics, the combined use of CBCT imaging and intraoral scanning allows the manufacturing of a 3D endodontic template. This template facilitates a straight access cavity to the root canal by guiding the endodontic bur to the exact area [17, 18]. The use of guided endodontics has been previously performed and reported in the literature as a safe [19] and predictable technique [20] which, in turn, leads to an improved long-term prognosis as they help to preserve the dental structure and avoid accidents such as deviations and perforations [18]. The present case report proposes a fully guided preparation as an attempt to minimize dentin loss and eliminate iatrogenic errors during fiber post removal.

## 2. Case Report

**Figure 1:**
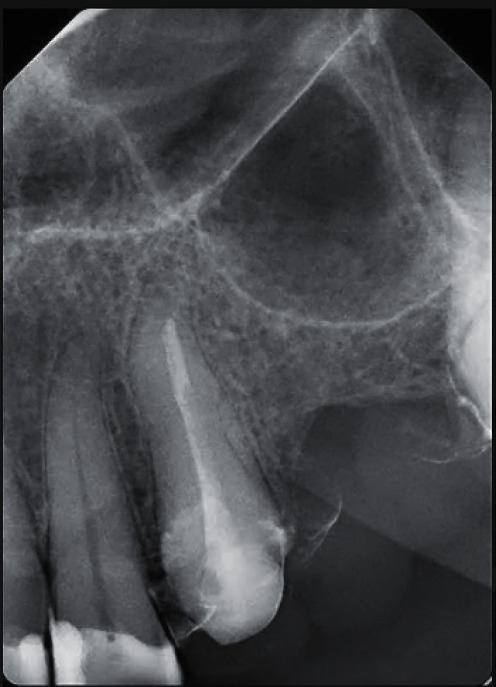
Preoperative radiographic assessment showing tooth #23 with permanent core retained with a fiber post and inadequate root canal filling.

**Figure 2:**
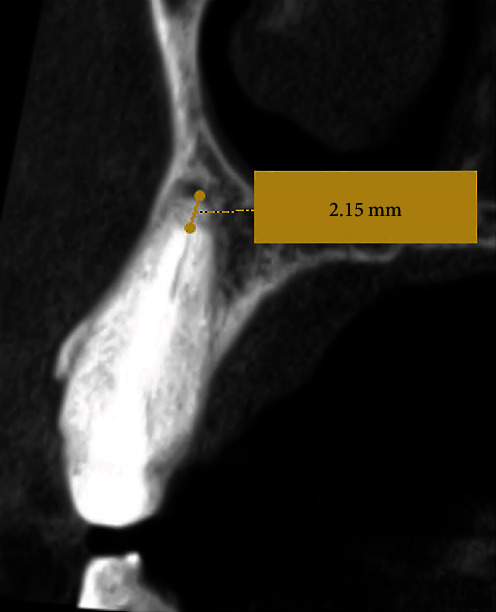
Preoperative CBCT assessment. Sagittal slice of tooth #23 confirms apical root resorption with the radiolucent area, 2.15 mm short obturation, and fiber post cemented up to the middle third.

A 40-year-old normal healthy male patient, ASA I (According to the American Society of Anesthesiologists), presented to the postgraduate endodontic clinic at King Abdulaziz medical city, complaining of discomfort in the upper left anterior area for the past month. After clinical examination, tooth #23 exhibited sensitivity to percussion while the mobility was normal (grade I) as tested using two ends of metallic instruments. The periodontal probing depths were checked with a periodontal probe and were found within normal limits. Radiographic interpretation revealed a permanent core retained with a fiber post of unknown sources with an inadequate root canal filling and periapical radiolucency (Figure 1). A CBCT was taken using Planmeca ProMax 3D S (Planmeca OY, Helsinki, Finland) operated at 80 kV, 3.0 mA, and voxel size of 0.15 mm to fully assess the anatomy of tooth #23 and surrounding structures. The imaging revealed apical root resorption and a radiolucent area with intact buccal and palatal plates. The obturation was found to be 2.15 mm short from the apex and the fiber post was located up to the middle third (Figure 2). Based on these, a diagnosis of a previously root canal-treated tooth with symptomatic apical periodontitis was reached. Among the various treatment modalities, nonsurgical root canal retreatment with fiber post removal using a conservative fully guided approach was proposed. The procedure's benefits and risks were explained to the patient, and consent was obtained.

**Figure 3:**
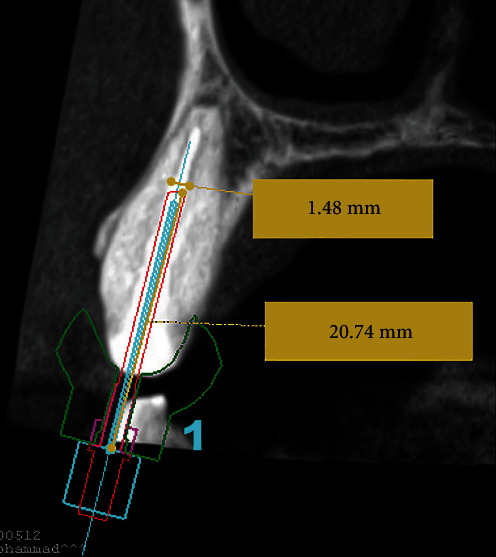
Virtual planning of fiber post removal. The drill was positioned along the long axis of the fiber post set up to its apical tip.

**Figure 4:**
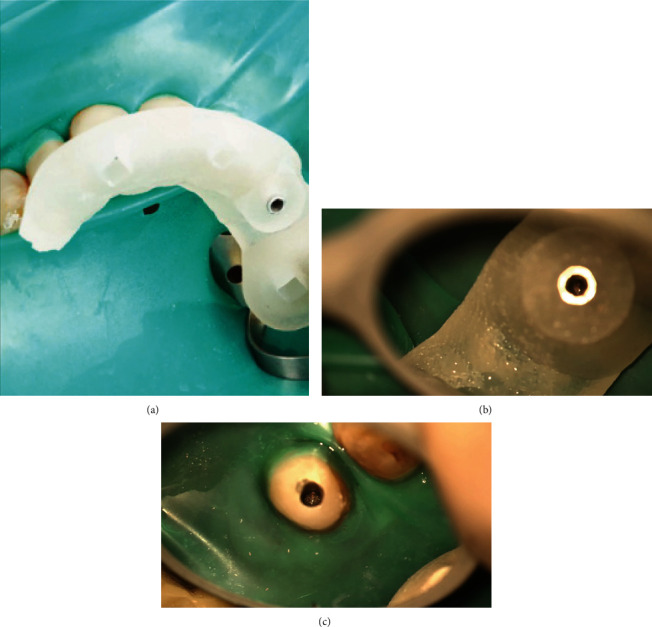
(a) Under rubber dam isolation, the guide was positioned on the teeth to check the correct fitting. (b, c) Intracanal space following complete removal of the fiber post with coronal gutta-percha exposure.

**Figure 5:**
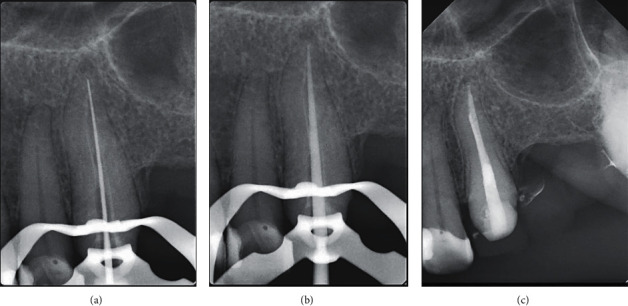
(a) Working length determination radiograph. (b) Master gutta-percha cone radiograph. (c) Final radiograph.

An impression of the upper arch was made using polyvinyl siloxane material (Imprint 4, 3 M, Saint Paul, Minnesota, USA) and poured to fabricate a diagnostic cast. The cast was then scanned by using the desktop laser scanner R700 Desktop (3Shape, Copenhagen, Denmark). Then, both the DICOM file from CBCT images and the cast surface scan file were merged using implant planning software (ProDigiDent, ImplaStation for Windows x6464 Bit Beta Version) and superimposed by selecting three reference landmarks in both files. The template was made with 3.5 mm thickness and 0.15 mm offset, which was extended to cross the midline for maximal stability. The drilling space was planned based on the location, diameter, and apical extent of the fiber post in the sagittal view. It was found to be 20.74 mm long with a 1.48 mm diameter apically. The space was virtually overlapped over the fiber post to drill through it with minimal dentin loss (Figure 3). The endo-guide template was then created and exported for printing using a digital light processing (DLP) (M-One; MAKEX Technology, Zhejiang, China) technology. A 3D printer (MiiCraft 125; MiiCraft, Jena, Germany) was used with a photo-polymerized biocompatible polymer resin (Freeprint Temp; DETAX GmbH & Co., Ettlingen, Germany) to print the template. The printer settings included 50 *μ*m thickness, 405 nm wavelength, and a curing time of 2.40 s per layer. To guide the bur in the created drilling space, a guiding sleeve with 3.0 mm external diameter, 1.7 mm internal diameter, and 5 mm length was virtually customized using CAD software (Google SketchUp) (SketchUp, Trimble Navigation, Sunnyvale, California, USA) and printed using a selective laser melting system (GE Additive company, Boston, MA, USA) with standard parameters. Both the custom sleeve and endo-guide template were integrated to fully guide the bur during the fiber post removal. In the second visit, the tooth was anesthetized using 2% lidocaine with 1:80,000 epinephrine (Lignospan Special; Septodont, Saint-Maur-des-fossés, France) and isolated with a rubber dam. The endo-guide template was fitted inside the patient's mouth (Figure 4(a)). After a satisfactory assessment of the fit and stability, with pumping movement, drilling was made with increments of 2 mm using a high-speed handpiece with a size 4 long-shank round bur (Thomas, Bourges, France), which has a 1.4 mm head diameter, 1.6 mm shank diameter and 28 mm shank length. The full procedure was performed by an endodontic resident and recorded under a dental operating microscope (ZEISS OPMI pico; Carl Zeiss Meditec AG, Oberkochen, Germany). The procedure took 14 min and 55 s to expose the coronal gutta-percha inside the canal (Figure 4(b) and 4(c)). Then, K3 rotary files (Sybron Endo, Orange, USA) sizes 25 06 taper and 30 06 taper were used along with chloroform to remove the old obturating materials. A size 30 K-file was then inserted to verify the working length with an electronic apex locator (Root ZX; J Morita, Tokyo, Japan) and confirm it radiographically (Figure 5(a)). Then, additional shaping and cleaning were done with ProTaper Next rotary files (Dentsply Sirona, Ballaigues, Switzerland) sizes X2 and X3, and 5.25% NaOCl irrigation, respectively. The root canal was then dried with paper points and obturated with gutta-percha and AH Plus sealer (Dentsply Sirona, Ballaigues, Switzerland) using the continuous-wave compaction technique. Finally, the tooth was temporarily restored using the double seal technique with zinc oxide and zinc sulfate-based temporary material (Cavit W; 3 M ESPE, St. Paul, MN, USA) and resin-modified glass ionomer material (Photac Fil; ESPE, Norristown, PA, USA) filling materials and referred for final restoration (Figure 5(b) and 5(c)).

## 3. Discussion

The present case report describes a guided technique for the removal of fiber post during nonsurgical endodontic retreatment using CBCT and a 3D printer. Evaluation of the preoperative periapical radiograph of tooth #23 confirmed the presence of a fiber post that extended to the middle third of the root. Overall, fiber posts can be removed using one or a combination of several techniques such as ultrasonic vibrations, drilling with long-shank burs, and using special post removal kits [8]. Nevertheless, currently used post removal techniques frequently result in procedural errors such as excessive removal of intraradicular dentin, deviation from the root axis, and perforation of the root structure [9]. The difficulty of post removal varies according to post type, design, material, length, and cementing material [5].

CBCT is a reliable and noninvasive tool that has gained widespread use in the diagnosis and treatment planning of dentoalveolar conditions. The American Association of Endodontists and the American Academy of Oral and Maxillofacial Radiology have published a joint position statement related to the use of CBCT [21]. The need for a CBCT scan can be considered if careful evaluation of differently angled periapical radiographs failed to yield conclusive information or if further information in the buccolingual dimension is still required. In cases deemed appropriate for the scan, a narrow field of view that is associated with reduced radiation dose and higher spatial resolution is advisable [21]. Hence, CBCT should only be used as an adjunctive tool in certain clinical situations such as assessment of teeth with suspected complex morphology, localization of obliterated canals, evaluation of the endodontic treatment outcome, and planning of nonsurgical and surgical endodontic retreatment as well as dentoalveolar trauma and resorptive defects [21, 22]. Furthermore, CBCT is frequently used in oral implantology for three-dimensional planning to quantify the alveolar bone levels and to localize vital anatomic structures [23] as well as in guided implant surgery to help with implant site preparation and implant placement [24].

Zehnder and his colleagues introduced the concept of “guided endodontics” to facilitate access cavity preparation for teeth with root canal obliteration and reported that deviations of planned and prepared access cavities were ranging from 0.17 to 0.47 mm at the tip of the bur, while the mean of angle deviation was 1.81° [18]. In this case, a custom sleeve was fabricated and integrated with the endo-guide template to adequately guide the drilling pathway without the risk of resin being damaged by over-heating or undesirable resin drilling [25]. Therefore, in comparison to guided implant surgery, the accuracy of guided endodontics is considered relatively high [26].

The guided preparation that was used to remove the fiber post in our study conserved as much tooth substance as possible. A previous study revealed that the mean amounts of prepared dentin in traditional and conservative guided approaches to access root canal systems were 49.9 and 9.8 mm^3^, respectively. Moreover, unlike traditional access preparations, the success of the guided approach is not influenced by the operator's experience [27]. Hence, the conservative guided technique significantly reduces access cavity size, follows a clear path, and preserves the tooth structure [28].

The guided approach presented in this case report has some limitations. For instance, the technique requires prior training for the clinician with an associated learning curve. Also, guided endodontic procedures require the use of CBCT to permit 3D evaluation of the target area. CBCT is associated with more ionizing radiation than conventional radiographs [15], which might be concerning for some patients. Moreover, the presented approach is sensitive to distortions or errors made during intraoral scanning, 3D virtual planning, and printing of the guide. Another limitation of guided endodontics is that it does not enable immediate intervention due to the need for CBCT imaging and intraoral scanning in advance.

The speedy progress of digital dentistry workflows, supported by the evolving technology, will continue to improve the accuracy of guided endodontics. This progress will give rise to the widespread implementation of this digitally supported technique in dental practice.

## 4. Conclusion

A guided endodontics template created with virtual planning facilitated complete removal of the fiber post with no iatrogenic errors observed and shortened treatment time. Furthermore, to produce predictable results, this approach does not necessitate specialized training or extensive clinical experience.
